# Dialyzer reuse: is it safe and worth it?

**DOI:** 10.1590/2175-8239-JBN-2019-0134

**Published:** 2019-09-02

**Authors:** Ashish Upadhyay

**Affiliations:** 1 Boston University School of Medicine Section of Nephrology BostonMA United States of America Boston University School of Medicine, Section of Nephrology, Boston, MA, United States of America.

Dialyzer reuse, the practice of using the same dialyzer for multiple hemodialysis treatments, has been in place since 1960s.^1^^,^^2^ While there has been a steady decline of dialyzer reuse in the United States and Europe since late 1990s, it continues to be mainstream across most of the developing world.^3^^-^6

Dialyzer reuse involves a complicated multistep process that includes rinsing, cleaning, performance testing, and disinfection of dialyzers prior to reuse. The process requires the use of cleaning and germicidal agents that are potentially toxic, and accidental contact with these agents may expose both patients and dialysis staff to health hazards.^1^^,^^7^^-^9 There are also reports of Gram-negative bacteremia outbreaks from the break-down in infection-control systems,^10^^-^12 and even a low-level exposure to toxins and microbiological contamination may contribute to chronic inflammation. Despite these potential risks, there are no randomized-controlled trials comparing single-use and reuse practices, and the evidence from observational studies are inconsistent.^6^^,^^13^ There is also a concern for conflicts of interest as studies sponsored by disinfectant manufacturers tend to show single-use and reuse practices having similar health outcomes while those sponsored by dialyzer manufacturers are more prone to show reduced risk with single-use.^13^^,^^14^ Notwithstanding the available evidence, given the 50 years of clinical experience with dialyzer reuse, there is a general agreement that the reuse process is likely safe when there is a strict adherence to the standards set by the Association for the Advancement of Medical Instrumentation (AAMI).^15^

Traditionally, dialyzer reuse was employed to improve dialyzer membrane biocompatibility, particularly of cellulose membranes, and lower the risk of first-use syndromes observed in ethylene oxide-sterilized dialyzers. These advantages of reuse are now moot due to the widespread availability of biocompatible dialyzer membranes and favorable sterilization techniques.^6^ Economic considerations, on the other hand, continue to make dialyzer reuse appealing for many dialysis service providers. Economic considerations, however, are not uniform around the world or, in many places, even within the same country. There is an argument that the cost-benefit with reuse may be negligible in areas of the world where the costs related to reuse-related personnel and safe storage space are high.^6^ The relative cost savings, however, is expected to be higher in areas where the personnel and space costs are low.^3^^,^^5^^,^^16^ Even a marginal cost-saving would be important in financially strained health systems, or in instances when patients share the cost of their dialysis care.

In this backdrop, Dr. Ribeiro and colleagues report the findings of their small cross-over study examining the differences in clinical and microbiological parameters with single-use and reuse practices.^17^ Ten patients were selected to undergo one hemodialysis treatment using the single-use practice and twelve hemodialysis treatments using the reuse-practice. Clinical, laboratory, and microbiological parameters were collected during the single-use treatment (N = 10 sessions) and during the 1^st^, 6^th^, and 12^th^ reuse treatments (N = 30 sessions). High-flux polysulfone dialyzers that were steam-sterilized were used, and the reprocessing was done manually using the institutional protocol that was based on the AAMI’s standards. Dialyzers were cleaned using the solution composed of peracetic acid, hydrogen peroxide, acetic acid, and active oxygen (Peroxide P50, Bell Type Industries, Brazil). Inflammatory biomarkers, C-reactive protein (CRP), and ferritin were noted to be high at baseline and increase after hemodialysis in both single-use and reuse treatments. Endotoxin levels were similar before and after both single-use and reuse treatments. Median serum levels of CRP and endotoxins, pre- and post- hemodialysis treatments, were not significantly different between single-use and reuse sessions. Blood and protein residues were found in most dialyzers after the reuse sessions, but samples from the sanitizing liquid stored in the dialyzer blood chamber were free of bacterial and endotoxin contamination.

While the findings from this study provide reassurances about the safety of dialyzer reuse, there are important caveats. First, there was no wash-out phase in the study, and patients were using reused dialyzers prior to their first and only single-use treatment. So, if there is any benefit to single-use dialyzer, one treatment alone may not be adequate to observe a change in clinical and laboratory parameters. Second, the adverse consequences from reuse tend to occur when there are human errors in the implementation of the AAMI’s standards. Therefore, a reassuring finding in a study of ten patients still leaves open the question of whether the reuse practice is safe in large health systems where any lapse in the execution of reprocessing standards may lead to adverse patient outcomes. Third, the findings from this study are only valid for the type of dialyzers and cleaning agents used in the study, namely steam-sterilized high-flux polysulfone dialyzers and the peracetic acid-based cleaning system. It would thus not be advisable to extrapolate these findings to modified-cellulose dialyzers, dialyzers that use sterilization practices other than steam, or to reuse systems that do not use peracetic acid-based cleaning agents.

In conclusion, the study by Dr. Ribeiro and colleagues reinforces the notion that the dialyzer reuse practice is likely safe when performed according to the standards set by the AAMI. The medical reasoning for dialyzer reuse, however, is obsolete in the current era of biocompatible dialyzers, and the potential for cost-saving is the only rationale for its continued practice. It is now imperative to conduct a systematic cost-benefit analysis of reuse practices in developing countries where any cost-saving can have an important impact in the availability of hemodialysis treatments.

## References

[1] Lacson E Jr, Lazarus JM. Dialyzer best practice: single use or reuse? Semin Dial 2006;19:120-8. 10.1111/j.1525-139X.2006.00137.x 16551289

[2] Upadhyay A, Sosa MA, Jaber BL. Single-use versus reusable dialyzers: the known unknowns. Clin J Am Soc Nephrol 2007;2:1079-86. 10.2215/CJN.01040207 17702728

[3] Dhrolia MF, Nasir K, Imtiaz S, Ahmad A. Dialyzer reuse: justified cost saving for south Asian region. J Coll Physicians Surg Pak 2014;24:591-6. 25149841

[4] Prasad N, Jha V. Hemodialysis in Asia. Kidney Dis (Basel) 2015;1:165-77. 10.1159/000441816 PMC4934815 27536677

[5] Cusumano A, Garcia GG, Di Gioia C, Hermida O, Lavorato C; Latin American Registry of Dialysis and Transplantation. The Latin American Dialysis and Transplantation Registry (RLDT) annual report 2004. Ethn Dis 2006;16:S2-10-3. 16774002

[6] Upadhyay A, Jaber BL. Reuse and Biocompatibility of Hemodialysis Membranes: Clinically Relevant? Semin Dial 2017;30:121-4. 10.1111/sdi.12574 28066932

[7] Vanholder R, Noens L, De Smet R, Ringoir S. Development of anti-N-like antibodies during formaldehyde reuse in spite of adequate predialysis rinsing. Am J Kidney Dis 1988;11:477-80. 10.1016/s0272-6386(88)80083-0 3376932

[8] Ng YY, Yang AH, Wong KC, Lan HY, Hung TL, Kerr PG, et al. Dialyzer reuse: interaction between dialyzer membrane, disinfectant (formalin), and blood during dialyzer reprocessing. Artif Organs 1996;20:53-5. 10.1111/j.1525-1594.1996.tb04418.x 8645130

[9] Schoenfeld PY. The technology of dialyzer reuse. Semin Nephrol 1997;17:321-30. 9241717

[10] Oyong K, Marquez P, Terashita D, English L, Rivas H, Deak E, et al. Outbreak of bloodstream infections associated with multiuse dialyzers containing O-rings. Infect Control Hosp Epidemiol 2014;35:89-91. 10.1086/674399 24334805

[11] Flaherty JP, Garcia-Houchins S, Chudy R, Arnow PM. An outbreak of gram-negative bacteremia traced to contaminated O-rings in reprocessed dialyzers. Ann Intern Med 1993;119:1072-8. 10.7326/0003-4819-119-11-199312010-00003 8239225

[12] Welbel SF, Schoendorf K, Bland LA, Arduino MJ, Groves C, Schable B, et al. An outbreak of gram-negative bloodstream infections in chronic hemodialysis patients. Am J Nephrol 1995;15:1-4. 10.1159/000168793 7872357

[13] Galvao TF, Silva MT, Araujo ME, Bulbol WS, Cardoso AL. Dialyzer reuse and mortality risk in patients with end-stage renal disease: a systematic review. Am J Nephrol 2012;35:249-58. 10.1159/000336532 22353780

[14] Lowrie EG, Ofsthun NJ. To reuse or not to reuse: a tale of 2 studies. Am J Kidney Dis 2006;47:372. 10.1053/j.ajkd.2005.11.021 16431272

[15] American National Standards Institute/Association for the Advancement of Medical Instrumentation (ANSI/AAMI). ANSI/AAMI RD: 2008/(R)2013 - Reprocessing of Hemodialyzers. Washington: ANSI/AAMI; 2013.

[16] Qureshi R, Dhrolia MF, Nasir K, Imtiaz S, Ahmad A. Comparison of total direct cost of conventional single use and mechanical reuse of dialyzers in patients of end-stage renal disease on maintenance hemodialysis: A single center study. Saudi J Kidney Dis Transpl 2016;27:774-80. 10.4103/1319-2442.185242 27424697

[17] Ribeiro IC, Roza NAV, Duarte DA, Guadagnini D, Elias RM, Oliveira RB. Clinical and microbiological effects of dialyzers reuse in hemodialysis patients. Braz. J. Nephrol 2019;24:pii: S0101-28002019005004103. 10.1590/2175-8239-JBN-2018-0151 PMC6788851 30720850

